# Confronting new challenges: Faculty perceptions of gaps in current laparoscopic curricula in a changing training landscape

**DOI:** 10.1016/j.sopen.2023.09.006

**Published:** 2023-09-12

**Authors:** Leslie Bernal Charondo, Riley Brian, Shareef Syed, Hueylan Chern, Jeannette Lager, Adnan Alseidi, Patricia O'Sullivan, David Bayne

**Affiliations:** a University of California, San Francisco, School of Medicine, San Francisco, CA, USA; b University of California, San Francisco, Department of Surgery, San Francisco, CA, USA; c University of California, San Francisco, Department of Obstetrics, Gynecology, & Reproductive Sciences, San Francisco, CA, USA; d University of California, San Francisco, Department of Urology, San Francisco, CA, USA

**Keywords:** Laparoscopic surgery, Surgical education, Simulation

## Abstract

**Background:**

Opportunities for residents to develop laparoscopic skills have decreased with the rise in robotic operations and the development of complex, subspecialized laparoscopic operations. Given the changing training landscape, this study aimed to identify laparoscopic surgeons' perceptions of gaps in current laparoscopic skills in general surgery, obstetrics-gynecology, and urology residency programs.

**Methods:**

Laparoscopic surgeons who operate with residents participated in semi-structured interviews. Questions addressed expectations for resident proficiency, deficits in laparoscopic surgical skills, and barriers to learning and teaching. Two authors independently coded de-identified transcripts followed by a conventional content analysis.

**Results:**

Fourteen faculty members from thirteen subspecialties participated. Faculty identified three main areas to improve laparoscopic training across specialties: foundational knowledge, technical skills, and cognitive skills. They also recognized an overarching opportunity to address faculty development.

**Conclusions:**

This qualitative study highlighted key deficiencies in laparoscopic training that have emerged in the current, changing era of minimally invasive surgery.

**Key message:**

This qualitative study identified laparoscopic educators' perceptions of deficiencies in laparoscopic training. Findings emphasized the importance of incorporating high quality educational practices to optimize training in the current changing landscape of laparoscopic surgery.

## Highlights

• Faculty identified multiple gaps in laparoscopic training across specialties.
• Simulation requires technical and cognitive skills built on foundational knowledge.
• Faculty development is essential for operative teaching and feedback in laparoscopy.

## Introduction

The field of laparoscopic surgery has changed significantly in recent years with the rise in robotic-assisted operations and the application of laparoscopy to more complex cases [[1], [2], [3], [4]]. As a result, opportunities for surgical residents to actively participate in these procedures and master laparoscopic skills have decreased [[5], [6], [7], [8]]. New time demands, super subspecialization, regulatory changes, and evolving service structures further challenge resident learning in the operating room (OR) [1,6,7,9,10]. These changes have been reported across general surgery, obstetrics-gynecology (OBGYN), and urology [[1], [2], [3], [4]].

Despite efforts to address these challenges and expand training, both faculty and graduated residents have identified decreased resident preparedness for laparoscopic surgery [11]. Program directors reported that despite having passed the Fundamentals of Laparoscopic Surgery (FLS), graduated residents could not atraumatically manipulate tissue (30 %), recognize anatomical planes (26 %), suture (56 %), independently perform a laparoscopic cholecystectomy (30 %), or operate for 30 unsupervised minutes during a major procedure (66 %) [5]. In another study, Fellowship Council (FC) program directors, current fellows, and recent fellow graduates cited laparoscopic needle positioning and suturing (78 %) and bimanual coordination during dissection and retraction (72 %) as skills in most need of improvement [11].

Given the significance and scope of this challenge, numerous prior authors have worked to prioritize simulation activities for surgical trainees and develop innovative techniques to improve training in laparoscopy [[12], [13], [14]]. However, a gap exists in understanding current perceptions of broader curricular design from faculty who operate extensively with residents. Indeed, most prior work has focused on relatively narrow aspects of the quite expansive challenges facing laparoscopic training; furthermore, most such work preceded the current era of widespread robotic-assisted operations and complex laparoscopy [14,15]. The changing landscape requires re-examination of current training and existing gaps in skills to reimagine residents' laparoscopic curricula.

This study aimed to identify laparoscopic surgeons' perceptions of gaps in current laparoscopic skills training for residents in general surgery, OBGYN, and urology programs. The results of this study will assist surgical educators in creating laparoscopic curricula aligned with today's training climate.

## Methods

### Approach, context, and research paradigm

In this qualitative study, laparoscopic surgeons from general surgery, OBGYN, and urology at the University of California, San Francisco (UCSF) participated in semi-structured interviews. Surgeons from the three disciplines practice across four diverse clinical sites (public county hospital, Veteran's Association hospital, and two separate university hospitals). General surgery and urology residents receive training in laparoscopy starting as first year residents during a single in-person simulation session. General surgery residents have an addiitonal seven to ten sessions during their second year of residency. OBGYN residents participate in quarterly simulation sessions throughout residency emphasizing a variety of skills, including basic laparoscopy focusing on the FLS tasks. Residents from all programs are provided home laparoscopic kits for asynchronous practice. Residents participate in laparoscopy in all years of residency, though exposure varies by resident.

We approached this study through an interpretive research paradigm. We chose this approach to identify participants' invaluable personal insights and highlight *how* and *why* while acknowledging our own perspectives as researchers with experience and interest in the area [16]. Throughout the process, we adhered to published guidelines on qualitative research [17]. This study was determined to be exempt by our institutional review board (IRB #21-33846/21-33384).

### Participants and sampling strategy

We purposively sampled abdominal laparoscopic surgeons to ensure diverse representation of specialties and experience. We focused on recruiting surgeons with teaching experience. We selected these surgeons based on group discussion. LBC emailed 24 faculty (thirteen from general surgery, eight from OBGYN, and three from urology) soliciting voluntary participation in a 45–60-min one-on-one video interview with three weekly reminder emails. We collected demographics on specialty, gender, and experience. Gender referred to the socially constructed roles, behaviors, and identities of female, male, and gender-diverse people.

### Interview guide

The authors developed a semi-structured interview guide using standard principles (Appendix 1) [16,18]. We asked faculty about their expectations for resident proficiency, resident deficits in laparoscopic skills, and barriers to learning and teaching. We solicited suggestions for curricular improvements. We asked how faculty members' experiences differed by level for early residents (post-graduate years 1–2), mid-level residents (post-graduate year 3), and senior residents (post-graduate year 4 and higher).

### Data collection

One author (LBC, a fourth-year medical student at the time of data collection) with training in interview techniques conducted two pilot interviews with authors DB and HC; DB, HC, and PO'S reviewed the pilot interview transcripts, provided feedback on the interview technique, and further refined the interview guide. We did not include pilot interviews in the final analysis. LC conducted all subsequent interviews over remote video (Zoom Video Communications, San Jose, CA). We obtained verbal informed consent prior to the interview. We audio-recorded, transcribed using artificial intelligence software (Otter.ai, Los Altos, CA), de-identified, and reviewed all transcripts for accuracy. LBC, RB, PO'S, HC, and DB met regularly to review transcripts concurrent to the interview process. Interviews continued until we reached information sufficiency for a conventional qualitative content analysis [[19], [20], [21]].

### Data processing and analysis

LC and RB independently reviewed the initial transcripts to generate coding categories and met with DB, PO'S, and HC to discuss individual findings and generate a preliminary codebook while remaining open to new codes [19]. LC and RB independently coded two transcripts using this codebook and met to reconcile discrepancies, refine the codebook, and discuss the codebook with HC, DB, and PO'S. Then, LBC and RB double-coded the remainder of the transcripts and reconciled discrepancies in a similar fashion. After ten interviews, no new gaps and no new codes were needed, thus reaching sufficient conceptual depth [19,20]. We continued with four additional interviews to confirm that we did not hear any new gaps. LC organized codes using Dedoose analytic software (Version 8.3.35, Los Angeles, CA: SocioCultural Research Consultants, LLC). LC and RB reviewed coded excerpts to categorize resident needs from the content analysis. LBC and RB continued iterative discussion of findings with the remaining authors over regular meetings.

### Researcher characteristics and reflexivity

We considered reflexivity throughout our data analysis process [22]. The interviewer and first coder (LBC) had minimal laparoscopic experience, allowing her to ensure clarification during interviews. The second coder (RB) had laparoscopic experience, which permitted the developement of appropriate codes. Our remaining investigator team brought diverse perspectives to enhance the trustworthiness of our results, allow for rich discussion about relevant themes and practical strategies for educators, and reduce bias as insider researchers.

## Results

**Fig. 1:**
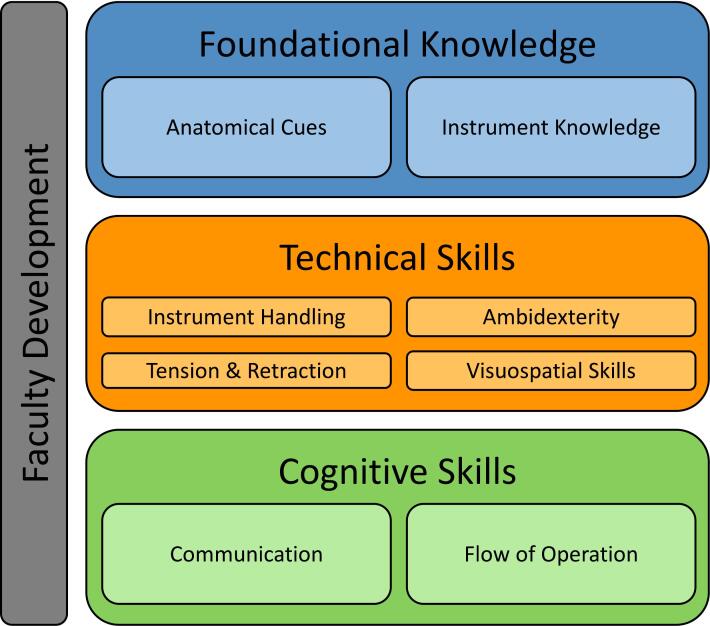
Faculty members identified gaps in resident laparoscopic skills and an overarching opportunity to address faculty development.

Fourteen faculty members participated in this study. Faculty represented thirteen subspecialties across three departments (eight from general surgery, three from OBGYN, and three from urology). Eight (57 %) identified as female and six (43 %) identified as male. Participants had 9 months to 33 years of experience (mean 9.9 years, SD 10.9) as faculty members and were 36 % (5/14) assistant professors, 43 % (6/14) associate professors, and 21 % (3/14) full professors, which is representative of the faculty overall. Interviews lasted 19 to 65 min (mean 32 min and 44 s). Faculty identified four main gaps in current laparoscopic skills training: foundational knowledge, technical skills, cognitive skills, and faculty development (Fig. 1). Gaps mentioned did not differ substantially by specialty, gender, or experience.

### Foundational knowledge

For this study, foundational knowledge was considered knowledge at the core of preparing individuals for surgical practice and includes knowing the facts, principles, and terminology that are essential to advancing learning in an academic discipline. Instrument and anatomic knowledge were two key factors identified under foundational knowledge.

#### Instrument knowledge

Faculty members felt that residents were knowledgeable about the basic function of most instruments except for the endostitch, laparoscopic needle drivers, and hook cautery. Faculty members also stated that residents were less informed about the principles of and indications for energy devices. One surgeon said, “*Frequently, they tell me I'm the only person that asked them about how energy devices work*… *They know that it works, but they don't understand*… *how it works. Once in a while, you can get into trouble with that” (P2)*. Varied instrument preference among providers performing the same procedure contributed to excess cognitive load among residents who were not well-versed in the range of instruments available in the OR: *“[Not knowing the different instruments] can be really hard because then you're focusing on what… kind of laparoscopic instrument you have, and not what you're supposed to be doing with it” (P7)*.

#### Anatomical cues

In contrast to their own residency training, participants felt current training had placed more focus on technical skill with less focus on surgical anatomy. Lack of surgical anatomy knowledge contributed to unsafe operations and injuries, particularly in the setting of distorted, inflamed, or bloody tissue.

“Basic understanding of anatomy on paper helps you with 3D relationships… particularly when the anatomy isn't so clear… That prevents you from cutting into things you shouldn't be cutting into” (P4)

### Technical skills

This study defined technical skills as sets of motor abilities required to perform laparoscopic tasks, such as suturing. Faculty identified gaps in four categories of technical skills: instrument handling, tension and retraction, ambidexterity, and visuospatial skills.

#### Instrument handling

Overall, faculty stated that entry-level residents were generally adept at camera handling, port placement, and instrument introduction, which was felt to be due to early exposure in previous years. Faculty noted deficits with the following laparoscopic instrument-dependent skills: suturing, providing retraction with instruments, and camera view optimization by adjusting the camera angle and/or position to maintain instruments in view. There were mixed faculty responses to resident performance in intracorporeal suturing and hook cautery. Faculty noted difficulty with laparoscopic suturing that challenged residents experienced with the robotic platform: “*suturing in a funny three dimensional setup is much easier with the robot*” (P2). Another respondent stated, “*I think learning laparoscopic skills without the robot is so much better than learning with the robot. It is transferable to the robot, it is not transferable from the robot to lap*” (P10). Faculty mentioned that residents' instrument handling improved if skills were practiced in simulation.

#### Tension and retraction

There were varied perceptions of mid-level residents' ability to provide retraction, tension, and compression of tissue without traumatizing it during dissection, particularly when attempting to optimize visibility. Many residents had yet to learn how to generate the appropriate amount of tension to maintain constant exposure for themselves or how to respond to tactile feedback from various tissue types. Faculty expressed confidence in residents' ability to use a sponge or laparoscopic peanut for retraction. Maintaining tissue tension based on visual and tactile feedback was mastered after extensive practice from a high volume of cases.

“The more junior ones would just grab the tissue and they want to retract, they just pull… [You can tell] how advanced [a resident] is by how delicately they can handle the tissue and they pull… just enough to do the work, but not more than that” (P2)

#### Ambidexterity

Even for the same surgical procedure, expectations for proficiency in performing two-handed laparoscopic procedures varied among faculty. For instance, one faculty member expected mid-level residents to perform a two-handed laparoscopic cholecystectomy, while another expected it only of senior residents. Regardless of expectations, all faculty stated that many residents were not using their non-dominant hand effectively during laparoscopic surgery.

#### Visuospatial skills

Unlike open surgery, laparoscopy requires translation of a three-dimensional (3-D) movement of the surgeons' arms outside of the patient's body to a two-dimensional (2-D) motion on the video display back to a 3-D action at the surgical site of interest. Often, residents' subtle hand movements translated to dramatic movements on the screen. Residents struggled with lack of depth perception, limited workspaces in the abdomen for pediatric or pelvic cases, and changes in orientation, such as in using the camera from a lateral port. Also, residents were challenged by positional awareness of instruments and anatomic relationships in the abdominal space, often leading to instrument collisions. Over time, residents developed “muscle memory” to simultaneously coordinate their movements outside and inside the patient's body.

“I think the main thing that people struggle with, particularly early on, is simply moving instruments inside and outside the body to [the] location that they want … Part of that is translating where you are in a 3D space, but also understanding how movements on the outside of the body translate to movements they see on the inside of the body” (P4)

“There's a lot of things … [that lead to] cognitive overload because it's a surgery where your eyes are actually not on your hands versus … an open procedure where [you] focus on your hand … [to] see the bigger picture … they just have to kind of rewire their brains a little bit” (P3)

### Cognitive skills

For this theme, we defined cognitive skills, such as flow of operation and communication, as mental processes used in the process of adapting, forward-planning, reasoning, and problem-solving.

#### Flow of operation

For most operations, there is a “script” of steps that guide the procedure. Faculty members noted that residents who had not reviewed the steps for an operation made the operative experience more challenging for those involved. In addition, there was variability among faculty members for a given procedure, which can create a challenge for junior learners. Participants stated that entry- and mid-level residents were often “married” to the standard procedural steps, lacked an understanding of diversion indications, and could not identify high-risk points. Faculty members remarked that when faced with an obstacle, residents were unable to reassess their progress and adapt without further guidance from the attending. As residents gained confidence, they learned to think ahead, anticipate the surgeon's next move, and adapt their approach to help the case move forward.

“When anatomy is normal, they know the steps … if you have distorted anatomy, they really struggle with trying to restore the normal anatomy” (P5)

#### Communication

All faculty respondents mentioned hurdles around feedback receptivity and the inability of residents to articulate their struggles. Communication was not always verbal – it was also described as a dance to explain the importance of communication and trust during a case – junior residents had to try to learn the steps and the rhythm of the dance with their “partner” while the chief resident was expected to teach the dance:

“So when they're junior, and kind of focused on how to manage both hands and less focused on how to coordinate movements between themselves and someone else” (P4)

Notably the subtext of our interviews revealed the importance of longitudinal exposure of faculty to trainee development, and the importance of sustained resident skill observation over time to develop cognitive and planning skills that are difficult to teach with communication exchanges spanning one-time or sporadic exposures.

### Faculty development

As trainees, many faculty were operating unsupervised at an early stage of their training - a remarkable contrast to their own level of comfort with resident autonomy attributed to lawsuits and patient safety. The majority of faculty acknowledged having limited formal training in providing feedback to residents on surgical techniques in the OR, especially with left-handed residents. They also noted difficulty knowing how to educate residents in the setting of so many techniques to teach.

“It's not enough hours in the day, especially now we have more and more different types of procedures that they haven't learned, used to be just open and a little bit of laparoscopic. Now we have endoscopy, now we have robotic surgery and there's just simply not enough time to teach them” (P3)

While operating with a struggling resident, faculty described difficulty providing verbal cues and instead taking control of instruments due to concerns about patient safety.

“It's actually very stressful to do a laparoscopic case with someone who doesn't 100% know what they're doing because you don't have control... my hands are really far away from their hands and none of our hands are actually on organs… I'm not sure how to help residents more than what I'm already doing. So, I don't know what kind of training I would need. But I'm sure that it would be helpful if I had it (laughs)” (P7)

Although faculty with positions in education had a greater understanding of residents' training, most faculty had limited knowledge of current laparoscopic training. Some faculty discussed expectations and surgical skill goals with residents. Most faculty expressed interest in engaging in development on providing feedback and teaching surgical skills in the OR.

“It would be really helpful if I knew where the residents were, because … I can help them progress further, instead of just reiterating what they've already have established… Some residents need a little bit more attention on some other things and not pushing them beyond what they're supposed to do” (P11)

## Discussion

This qualitative study identified crucial areas where there are ongoing gaps in laparoscopic training in general surgery, OBGYN, and urology: foundational knowledge, technical skills, cognitive skills, and faculty development (Fig. 1). Our needs assessment presents concrete suggestions for educators who endeavor to improve laparoscopic training for surgical residents.

Prior work has reported on the challenges of mastering the complexities of laparoscopic surgery [23,24]. Before achieving proficiency, surgeons performing laparoscopic surgery experience more blood loss, higher complication rates, longer operative times, and greater patient length of hospital stay [25]. Unfortunately, others have suggested that gaining adequate experience in laparoscopy has become more difficult due to the rising dominance of robotic surgery and the increasing complexity of laparoscopic cases [[1], [2], [3], [4]]. Our findings support this challenge. Clearly, there is room for improved training to facilitate earlier and more robust laparoscopic skill acquisition. Many authors have described relevant, effective, and evidence-based interventions [26,27]. Numerous such interventions focus on laparoscopic simulation, which has consistently been shown to improve trainee performance [28,29]. Other proposed strategies to enhance the training experience include instituting mandatory and protected time for practice and establishing a comprehensive curriculum with diverse teaching models [30,31]. Off-site training with remote or peer-based feedback offers an accessible option for laparoscopic skills acquisition from anywhere [[32], [33], [34], [35], [36], [37]]. Still others have proposed integration with robotic surgical training and an emphasis on shared skills [38]. These represent just a fraction of published interventions.

However, though we have implemented these and other interventions at our own institution, gaps in laparoscopic training persist. This highlights that in our contemporary complex training environment, the solution does not lie simply in more interventions, but in focusing on interventions' quality and the curriculum more holistically. After reviewing the literature and our findings, we propose strategies to implement in training curricula for all disciplines to enhance skill development (Table 1). Strategies may include introducing laparoscopic instruments early in training, using both low-cost and high-fidelity models depending on the skill being taught, incorporating cooperation and peer teaching, and explicitly discussing decision making processes. In many laparoscopic simulation exercises, residents are asked to individually practice a specific task with one set of instruments. In a new laparoscopic curriculum, we must optimize practice across competencies and promote trainee development through the stages of skill acquisition [39]. For example, a simulated laparoscopic bowel anastomosis can outline the foundational knowledge underlying instruments and anatomy, incorporate two residents to promote team communication, and provide multiple possible instruments to replicate decision-making in the OR environment. Similarly, simulations focusing on intracorporeal suturing can emphasize this technique as part of broader procedures (e.g., enteroenterostomy, sacrocolpopexy, ureteroureterostomy) and allow for progression from a low-cost dry model to a high-fidelity tissue model. Laparoscopic surgeons should attend such sessions, and we must incorporate training on peer and faculty feedback in the simulation sessions given our and others' findings around faculty development and feedback [40,41].

**Table 1:** Suggestions for laparoscopic skill simulation to address gaps in laparoscopic training in the areas of foundational knowledge (F), technical skills (T), and cognitive skills (C).

Suggestion	Steps to implement suggestion	Gap addressed by each step	Gaps addressed
Introduce instruments early in training	1. Perform a faculty survey of most used instruments in the operating room and incorporate this set of instruments into laparoscopic simulations 2. Instruct residents about using instruments with energy 3. Encourage residents to use instruments with both hands	1. “They're all a little different. That can be really hard because then you're focusing on what… kind of laparoscopic instrument you have, and not what you're supposed to be doing with it” (P7) 2. “Especially my junior residents don't actually understand how we use energy” (P2) 3. “Using their left hand to do anything useful they struggle a lot with” (P9)	F- Instrument knowledge T- Instrument Handling T- Ambidexterity
Use low-cost models for basic skills	1. Provide low cost models to reinforce instrument handling and understanding of the visuospatial environment of laparoscopy	1. “Oftentimes, the exposure that people rely on is in the operating room, when it doesn't necessarily need to be… a lot of the basic stuff could be done outside the operating room” (P4)	T- Instrument Handling T- Visuospatial Skills
Integrate high-quality models for advanced skills	1. Incorporate high-quality models, such as those with tissue, for more advanced skills 2. Encourage the use of high-quality models to understand tension and retraction, practice ambidexterity, progress in visuospatial ability, and respond to anatomical cues 3. Promote cognitive understanding of operational flow by enabling residents to conduct an entire operation using high-quality models	1. “I think it's easy to practice, like intracorporeal tying, because you can do that in a box. But then being able to practice, like, the different and varying anatomies… is a lot harder” (P11) 2. “Things like seeing tissue planes and like tissue tension and things like that … I don't think things like working on a box are going to help. I think that you need like real tissue or something that is simulating real tissue” (P13) 3. “You're focused on technical tasks, but that's only about 5 % of the operation” (P9)	F- Instrument knowledge F- Anatomical Cues T- Tension and Retraction T- Ambidexterity T- Visuospatial Skills C- Flow of operation
Incorporate cooperation and peer teaching into simulation	1. Emphasize the importance of cooperation, communication, and team work during all simulation exercises 2. Pair residents during laparoscopic simulation to facilitate practice in assisting and peer feedback 3. Train residents using a “train the trainer” curriculum to promote peer teaching	1. “The two people were not coordinated at all” (P4) 2. “I think what they really struggle with is assisting. I think it's really hard for them to know how to assist somebody else” (P5) 3. “The fellow was trying to instruct the junior resident what to do, but they were not communicating. So they're retracting in the wrong location, not exposing for each other. It's kind of two people dancing with but all of them have left feet” (P4)	C- Flow of operation C- Communication
Describe decision-making	1. Provide narrated video resources that incorporate both visual guidance and oral reflection of decisions being made 2. Encourage residents to review videos prior to simulation exercises and operations	1. “One of the good ways to teach is to watch a video… but watch the video with somebody around who actually knows what they're doing… so that you don't have a lot of wasted time learning” (P2) 2. “I think one of the things that can be challenging is… if someone is like, clearly unprepared for the case” (P1)	F- Anatomical Cues C- Flow of operation C- Communication

This study has limitations. The findings of this single-institution qualitative report may not translate across all contexts. However, we hope that the results of this study might provide insight into challenges faced in the current laparoscopic training era experienced by others involved in surgical education. The faculty in this study do not conduct all types of laparoscopic procedures, and thus the strategies proposed in this study may not apply universally. Similarly, the diverse specialties of the represented faculty limit the application of results to any specific field. Additionally, we did not explore the perspectives of curriculum developers in this study. Future studies could harness this study's findings to update simulation-based curricula and examine the impact of such curricula on resident surgical competency and proficiency. Additional work might investigate the resident perspective of curricular gaps. While prior work has assessed trainees' views of specific procedures or curricula, there has not been substantial study of trainees' broader perceptions of current laparoscopic training [42,43]. Other future work could ensure that updated laparoscopic curricula meet the needs of all parties involved in laparoscopic skills training.

## Conclusions

Faculty identified gaps in laparoscopic training in the current changing training landscape that current laparoscopic curricula are not addressing. Our study contributes to the existing literature by adding a broad and educator-centered perspective on the known laparoscopic skill deficiencies. We have highlighted the importance of rethinking laparoscopic training and have provided recommendations for redesigning laparoscopic curricula.

## Funding sources

This work was supported by the UCSF Academy of Medical Educators Innovations Funding for Education 2022. Riley Brian receives funding for his research through the Intuitive-UCSF Simulation-Based Surgical Education Research Fellowship.

## Ethics approval

This study was determined to be exempt by our institutional review board (IRB #21-33846/21-33384).

## CRediT authorship contribution statement

**Leslie Bernal Charondo:** Conceptualization, Formal analysis, Investigation, Methodology, Writing – original draft, Writing – review & editing. **Riley Brian:** Conceptualization, Formal analysis, Funding acquisition, Investigation, Methodology, Writing – original draft, Writing – review & editing. **Shareef Syed:** Conceptualization, Investigation, Methodology, Supervision, Writing – review & editing. **Hueylan Chern:** Conceptualization, Formal analysis, Funding acquisition, Investigation, Methodology, Supervision, Writing – review & editing. **Jeannette Lager:** Conceptualization, Investigation, Supervision, Writing – review & editing. **Adnan Alseidi:** Conceptualization, Investigation, Methodology, Supervision, Writing – review & editing. **Patricia O'Sullivan:** Conceptualization, Formal analysis, Funding acquisition, Investigation, Methodology, Supervision, Writing – review & editing. **David Bayne:** Conceptualization, Formal analysis, Funding acquisition, Investigation, Methodology, Supervision, Writing – review & editing.

## Declaration of competing interest

The authors have no relevant financial or non-financial interests to disclose.

## Appendix 1 Faculty interview guide

Thank you for agreeing to participate in this study about your experience as a faculty educator. Most of the interview will focus on laparoscopic procedures, but we will also ask about robotic procedures towards the end. You are free to skip any questions at any time in the interview and to stop the interview if you wish. Your responses will be transcribed and stored with a code number which is designed to protect confidentiality. All comments used in a report will not be identifiable.

Do you have any questions before we begin the interview?

This is [name] interviewing Participant # XX on [date].

1. What kind of surgeries do you perform? What PGY level residents do you work with?
  • Please describe the distribution of procedures between lap vs endoscopic vs robotic vs open.
2. What are your expectations of technical skills in _____ (insert case mentioned in previous question) for each PGY level with whom you work?
  a. What technical skills in ______ procedure do residents at each PGY level perform well?
  b. What technical skills in ______ procedure do residents at each PGY level struggle with?
3. Can you share an example of a successful teaching experience with a typical case?
  • What technical skills were the easiest for residents to learn?
4. Can you share an example of a challenging teaching experience with a resident in the OR?
  • Why was that skill difficult?
5. What is your understanding of the existing training curriculum outside of the OR for residents in the department?
6. What are gaps in resident laparoscopic training in your specialty?
  a. How could we better prepare residents in the skills you previously mentioned?
  b. What are the specific skills they should be practicing in simulation at home or at skills lab?
7. Have you received formal training in teaching and providing feedback in the operating room?
  a. Have you reached out to any colleagues for advice or suggestions on teaching in this setting?
8. How do your expectations for robotic procedures compare to laparoscopic? (If applicable to surgeon)

Demographics:

What is your gender?

How long have you been on faculty?

What is your rank (instructor, assistant professor, associate professor, professor)?

What department/division are you in?

Are you subspecialized? If so, in what?
